# Massive right hemothorax as the source of hemorrhagic shock after laparoscopic cholecystectomy - case report of a rare intraoperative complication

**DOI:** 10.1186/1754-9493-5-12

**Published:** 2011-05-19

**Authors:** Rapicetta Cristian, Paci Massimiliano, Ricchetti Tommaso, Tenconi Sara, Biolchini Federico, Belluzzi Emilio, Sgarbi Giorgio

**Affiliations:** 1 Thoracic Surgery Unit, Arcispedale Santa Maria Nuova, Viale Risorgimento 80, 42100 - Reggio nell'Emilia, Italy; 2 Division of General Surgery, Magati Hospital, Via Martiri della Libertà 6, 42019 - Scandiano (Reggio nell'Emilia), Italy

## Abstract

A 62-year old man was referred to our institution in hemorrhagic shock after a laparoscopic cholecystectomy for acute cholecystitis, performed at an outside hospital. A chest X-ray revealed a right-sided massive pleural effusion. Urgent surgical exploration was performed through a video-assisted mini-thoracotomy which revealed active bleeding from a pleural adherence. Successful hemostasis was achieved intraoperatively and the patient had an uneventful recovery. In absence of intra-abdominal hemorrhage, a hemothorax should be considered as a potential source of major bleeding in patients who develop symptoms of hypovolemia after laparoscopic surgery.

## Background

Although laparoscopic cholecistectomy (LC) is a well-established surgical procedure, a high index of suspicion should be maintained towards both surgical (injury during blind trocar insertion, unrecognized diaphragmatic lesions) and anaesthetic complications (gas embolism, extraperitoneal insufflation and surgical emphysema, pneumothorax and pneumomediastinum) [1]. A case of hemothorax complicating LC is here reported with no evident technical intra-operative problems nor abdominal complications.

## Case presentation

A 62-yrs old male patient was referred to our Institution due to massive right pleural effusion with severe hypovolemia at the end of VLC.

The procedure, planned for cholelithiasis, was performed in a peripheral hospital in about 2 hours and half, due to the presence of severe cholecistitis with empyema and massive intraperitoneal visceral adhesions. During the intervention pneumoperitoneum was kept constant at 12 cmH_2_O and no increase of the pressure was needed. Intra-operative bleeding was about 300 cc because of a difficult isolation of the fellea from the liver, but it was easily controlled by argon beam coagulator. No impairment of respiratory parameters was observed by the anaesthesiologist.

At the end of procedure, before evacuation of pneumoperitoneum, the patient developed a hypotension which was initially responsive to fluid administration, but quickly deteriorated after the weaning resulting in severe hypovolemic shock with worsening of respiratory parameters (tidal volumes, peak pressures and blood gases).

**Figure 1:**
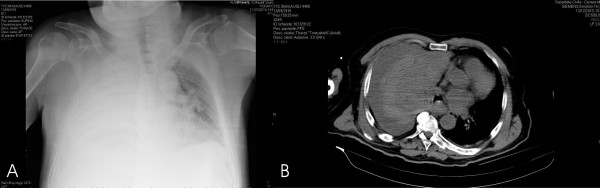
**A-B. Preoperative chest X-rays and CT-scan showing massive right pleural effusion with complete atelectasis of the lung**.

A chest X-ray performed in the operating theatre revealed a white right hemithorax, suggestive for massive peri-operative pleural effusion, that was soon confirmed by a contrast enhanced chest CT-scan (Figure 1A-B).

Considering the clinical and radiological findings, we decided to perform urgent surgical exploration through right video-assisted muscle-sparing lateral mini-thoracotomy in the 7^th ^intercostal space. A total amount of 2400 of fluid and clotted blood was evacuated from pleural cavity, and active bleeding became evident from a vascularized adherence between parietal pleural and right diaphragm located nearby anterior costophrenic angle. After haemostasis with bipolar electrocautery, accurate inspection of pleural cavity did not reveal tears of parietal pleura, diaphragm, internal mammary and intercostals vessels nor rib fractures.

**Figure 2:**
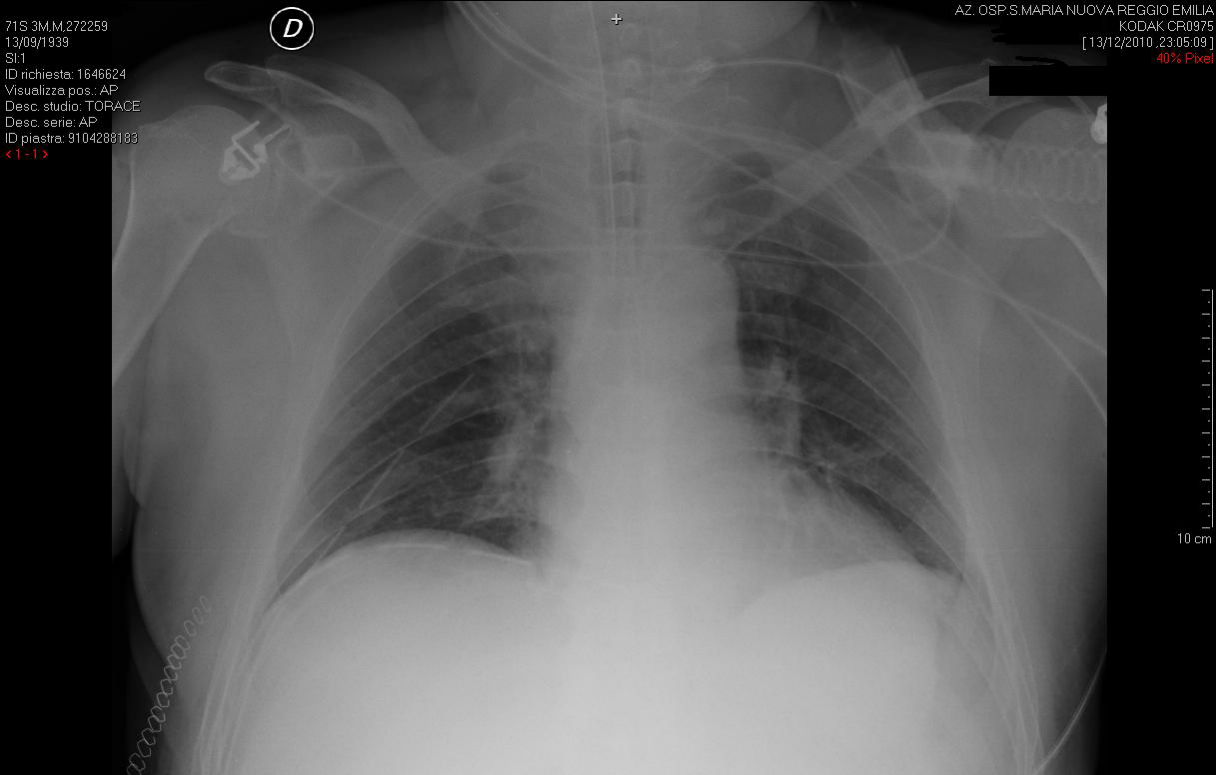
**Postoperative chest X-ray showing complete lung re-expansion**.

The post-operative course was uneventful with complete lung re-expansion (Figure 2). Chest drainage tubes were removed on 3^rd ^and 6^th ^post-operative day, respectively.

## Discussion

Thoracic bleeding is a rare complication of elective abdominal surgery, mainly resulting from not recognized, iatrogenic diaphragmatic tears. In this case, a pleural effusion usually appears post-operatively and progressively increases, but rarely this condition leads to severe hypovolemia and shock, unless a diaphragmatic artery has been injured.

Non-surgical related cause of intrathoracic bleeding could be intra-operative pneumothorax, due to extraperitoneal gas insufflation or prolonged mechanical ventilation, with high peak pressures: lung collapse can produce tension and rupture of vascularised adherences between parietal and visceral pleura. However, in this case, worsening of respiratory parameters (high ventilatory resistances, low tidal volumes) always anticipates signs of hypovolemia (tachycardia, hypotension, shock), resulting in an haemo-pneumothorax.

In this case, it has been supposed that laceration of the parieto-diaphragmatic adherence could have originated from diaphragmatic position change during pneumo-peritoneum, as it has been demonstrated in animal models [2]. Video-assisted thoracoscopic surgery (VATS) is a well-established technique in acute or delayed setting in traumatized patients with persistent bleeding, air leak, retained haemothorax or empyema [3]. Most Authors recommend that patients should be clinically stable to undergo VATS instead of thoracotomy [4-6].

## Conclusion

Intrathoracic bleeding is an extremely rare complication after abdominal surgery but it should be suspected and promptly diagnosed (through a chest X-Ray or Eco-fast) in case of worsening hemodynamic and respiratory failure in intra-operative or post-operative course. Medical supportive care only delays diagnosis leading to life-threatening conditions. Post-contrast TC is mandatory in order to exclude major vessels bleeding and thoraco-abdominal injury. We believe that a video-assisted mini-thoracotomy is a valid compromise even in these patients with hypovolemia because it allows rapid evacuation, inspection of pleural cavity and hemostasis, combining them with short and long-term benefits (less pain and analgesic consumption, shorter hospital stay, faster recovery) [7].

## Consent

Written informed consent was obtained from the patient for publication of this case report and any accompanying images. A copy of the written consent is available for review by the Editor-in-Chief of this journal.

## Competing interests

The authors declare that they have no competing interests.

## Contributions

RC performed thoracic surgical procedure and wrote the paper, PM and RT participated in surgical procedure, TS and BF reviewed literature, BE and SG reviewed the paper. All Authors read and approved the final manuscript.

## References

[B1] Cunningham AJ Anesthetic implications of laparoscopic surgery Yale J Biol Med 1998 71 6 551 78 10604786 PMC2578944

[B2] Sánchez-Margallo FM Moyano-Cuevas JL Latorre R Maestre J Correa L Pagador JB Sánchez-Peralta LF Sánchez-Margallo JA Usón-Gargallo J Anatomical changes due to pneumoperitoneum analyzed by MRI: an experimental study in pigs Surg Radiol Anat 2010 10.1007/s00276-010-0763-9 21181160

[B3] Abolhoda A Livingston DH Donahoo JS Allen K Diagnostic and therapeutic video assisted thoracic surgery (VATS) following chest trauma Eur J Cardiothorac Surg 1997 12 3 356 60 10.1016/S1010-7940(97)00192-9 9332911

[B4] Ahmed N Jones D Video-assisted thoracic surgery: state of the art in trauma care Injury 2004 35 5 479 89 10.1016/S0020-1383(03)00289-4 15081325

[B5] Manlulu AV Lee TW Thung KH Wong R Yim AP Current indications and results of VATS in the evaluation and management of hemodynamically stable thoracic injuries Eur J Cardiothorac Surg 2004 25 6 1048 53 10.1016/j.ejcts.2004.02.017 15145008

[B6] Wong MS Tsoi EK Henderson VJ Hirvela ER Forest CT Smith RS Fry WR Organ CH Jr Videothoracoscopy an effective method for evaluating and managing thoracic trauma patients Surg Endosc 1996 10 2 118 21 8932611 10.1007/s004649910028

[B7] Ben-Nun A Orlovsky M Best LA Video-assisted thoracoscopic surgery in the treatment of chest trauma: long-term benefit Ann Thorac Surg 2007 83 2 383 7 10.1016/j.athoracsur.2006.09.082 17257954

