# Correction: Massive right hemothorax as the source of hemorrhagic shock after laparoscopic cholecystectomy - case report of a rare intraoperative complication

**DOI:** 10.1186/1754-9493-7-20

**Published:** 2013-06-05

**Authors:** Cristian Rapicetta, Massimiliano Paci, Tommaso Ricchetti, Sara Tenconi, Federico Biolchini, Emilio Belluzzi, Giorgio Sgarbi

**Affiliations:** 1 Thoracic Surgery Unit, Arcispedale Santa Maria Nuova, Viale Risorgimento 80, – Reggio nell’Emilia 42100, Italy; 2 Division of General Surgery, Magati Hospital, Via Martiri della Libertà 6, – Scandiano (Reggio nell’Emilia) 42019, Italy

## Correction

After publication of this work [1], it was noticed that first and second names of co-authors of the article were reported in the wrong order with the exception of the corresponding author. The correct author list is listed in this article.

## Competing interests

The authors declare that they have no competing interests.
